# Acute and Chronic Toxicity of Carbamazepine on the Release of Chitobiase, Molting, and Reproduction in *Daphnia similis*

**DOI:** 10.3390/ijerph16020209

**Published:** 2019-01-13

**Authors:** Huihui Chen, Xiaohong Gu, Qingfei Zeng, Zhigang Mao

**Affiliations:** State Key Laboratory of Lake Science and Environment, Nanjing Institute of Geography and Limnology, Chinese Academy of Sciences, Nanjing 210008, China; hhchen@niglas.ac.cn (H.C.); qfzeng@niglas.ac.cn (Q.Z.); zgmao@niglas.ac.cn (Z.M.)

**Keywords:** carbamazepine, molting, *Daphnia similis*, chitobiase, chronic toxicity, reproduction

## Abstract

As one of the most frequently detected pharmaceutical compounds in aquatic environments, carbamazepine (CBZ) has recently been shown to cause acute and chronic toxicity in a variety of non-target aquatic organisms. However, little is known about the ecotoxicological effects it has on the molting and reproduction of crustaceans. The aim of the present work was to evaluate the acute and chronic toxic responses to CBZ in the crustacean *Daphnia similis*. After acute exposure (4 days), CBZ did not cause lethal toxicity at the tested concentrations. However, CBZ did inhibit the molting and release of chitobiase at concentrations higher than 6.25 μg/L, with 96 h EC_50_ (median effective concentration) values of 864.38 and 306.17 μg/L, respectively. The results of chronic exposure showed that the mean number of molts, size of the first brood, mean number of offspring per brood, mean number of broods per female, and total offspring per female decreased significantly with increasing CBZ concentrations. Significant effects of CBZ on the molting or fecundity in *D. similis* were observed even at concentrations as low as 0.03 μg/L. In conclusion, CBZ can cause inhibition of molting, delayed reproduction, and reduced fecundity in *D. similis*. CBZ toxicity to *D. similis* depends on the timing and duration of the exposure. Moreover, our results indicated that CBZ would act as an endocrine disrupter in *D. similis*, as with vertebrates (e.g., fish).

## 1. Introduction

In recent years, with advances in environmental analysis technology, a new class of environmental pollutants (pharmaceuticals and personal care products (PPCPs)) has begun to receive widespread attention [1,2]. Most PPCPs in the environment have low concentrations, complex structures, and difficult degradation and accumulation characteristics [3]. Although the concentrations are low in the environment, long-term pollution of PPCPs may cause endocrine disruption or reproductive toxicity to aquatic organisms, induce changes in biochemical functions of aquatic habitats, and do great harm to the environment [4,5,6,7,8,9,10]. Carbamazepine (CBZ), a heavily used pharmaceutical, is mainly employed for the treatment of epilepsy, arrhythmia, depression, and other diseases [6]. CBZ is discharged into the environment in a variety of ways, and can be residual in the environment, resulting in its persistence in water bodies and adverse effects on ecosystems [9]. CBZ contamination has been found in ng/L to μg/L concentrations in sewage influent and effluent water, surface water, and even drinking water [11,12,13,14,15]. For example, the concentration range of CBZ in the Shanghai sewage treatment plant was 230–1110 ng/L, and the maximum concentration in the Yangtze River was 1090 ng/L [11]. Liu et al. [13] also found that the detection rate of CBZ in the rivers in Nanjing was 100%, with a concentration range of 0.2–6.9 ng/L in the water, and 0.05–1.6 ng/g in the bodies of fish. In a recent study conducted at Taihu Lake, China, CBZ was the most frequently detected compound, with a detection rate of 100% in water, with concentrations ranging from 0.24 to 8.74 ng/L, and a 32% detection rate in biotic samples, including common carp (*Cyprinus carpio*), yellow catfish (*Pelteobagrus fulvidraco*), and crucian carp (*Carassius auratus*) [14].

Due to its high concentrations in aquatic environments, CBZ has received growing attention as an emerging contaminant based on its potential threat to non-target species [15,16,17,18]. For example, Malarvizhi et al. [15] reported that the median lethal concentration (LC_50_) of CBZ to the common carp (*Cyprinus carpio*) for 24 h was 59.70 mg/L, and CBZ induced alterations in the activities of glutamate oxaloacetate transaminase (GOT), glutamate pyruvate transaminase (GPT), and lactate dehydrogenase (LDH) in various organs. Aguirre-Martínez et al. [17] also reported that CBZ had significant effects on stable lysosome membrane, dibenzyl fluorescent dehydrogenase, glutathione S-transferase, glutathione peroxidase, lipid peroxidation, and DNA adducts of the common crab *C. carpio*. In addition, exposure to environmentally relevant concentrations of CBZ altered the siphoning behavior, biomarkers, *hsp* mRNA levels, and protein levels in the gills and digestive gland of the Asian clam (*Corbicula fluminea*). The changes in the biomarkers suggest that the effect of CBZ is related to oxidative stress [6]. Therefore, research into the toxic effects of CBZ on aquatic organisms should not be neglected. Concerning crustaceans, the existing knowledge about the toxic effects of CBZ is limited to the model species (e.g., *Daphnia magna*) [19,20]. For example, 1 μg/L CBZ can enhance reproduction and increases positive phototactic behavior in *D. magna* [19]. Kovacevic et al. [20] also reported that aromatic amino acids, including serine, glycine, and alanine, are potential bioindicators for sub-lethal CBZ exposure that may have altered energy metabolism. Molting is a natural biological process in arthropods, including crustaceans. During molting, the animal generates a new exoskeleton and sheds the old one to grow and develop [21]. In terms of endocrine-disrupting effects, molting is used as an effective biomarker to assess the toxicity of contaminants in crustaceans. Previous studies have reported that various xenobiotics (such as polychlorinated biphenyls, perfluorooctane sulfonate, pesticides, and heavy metals) can affect molting in crustaceans by disrupting molting hormone signaling, including chitinolytic enzyme, 20-hydroxyecdysone, and molting hormone signaling genes [22,23,24,25,26]; however, as a neuro-active pharmaceutical, and a potential endocrine disruptor in fish [10], neither the effects of CBZ on molting in crustaceans nor the underlying mechanisms are well studied or understood.

Thus, the aim of the current study was to determine the acute and chronic effect of CBZ on the molting, growth, and reproduction of *Daphnia similis*, which is a main crustacean species in Lake Taihu, China. As described above, CBZ is one of the most frequently detected pharmaceuticals in Lake Taihu, China [12,14], however, the potential toxicity of CBZ to *D. similis* is unknown. In the present study, newborn *D. similis* (<24 h old) were exposed to environmentally relevant concentrations of CBZ to investigate the effects of acute (4 days) and chronic (21 days) exposures on the survival, molting, growth, and reproduction of this species. After the acute exposure, the survival, number of molts, and chitobiase activity were measured. Chitobiase, a major chitinolytic enzyme in the molting fluid, is required for complete degradation of the old exoskeleton [21]. Based on the toxicological findings, we discuss the possible mechanism of CBZ action on molting and reproduction in this crustacean species.

## 2. Materials and Methods

### 2.1. Chemicals

**Figure 1:**
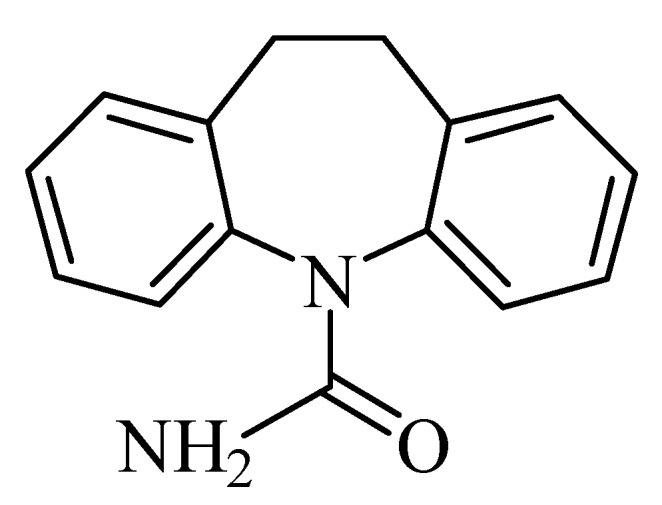
The structure of carbamazepine.

CBZ (CAS: 298-46-4, purity > 97%, Figure 1) and dimethylsulfoxide (DMSO) was purchased from Aladdin Industrial Corporation (Shanghai, China). A CBZ stock solution (10 mg/mL) was prepared in DMSO and stored in brown bottles. CBZ stock solution was made anew every week.

### 2.2. Test Organism

*D. similis* organisms were isolated from Lake Taihu (Wuxi, Jiangsu, China) and had been cultured and maintained in our laboratory for one year. The animals were cultured in 200-mL beakers at 20 ± 1 °C under a light-dark period of 16:8 h and fed with green algae (*Scenedesmus obliquus*; 10^6^ cells/mL) according to the Organization for Economic Cooperation (OECD) test guidelines 202 and 211 [27,28]. The green algae *S. obliquus* was cultured axenically in a liquid M4 medium [27], under the same conditions as the animals. Newborn *D. similis* (<24 h old) were used in the experiment for the acute and chronic exposure. 

### 2.3. Experimental Design

#### 2.3.1. Acute Exposure

The acute toxicity tests (4 days) were performed following the OECD test guideline 202 [27]. In accordance with the results of previous literature [19,20], six nominal concentrations of CBZ treatments (6.25, 12.5, 25, 50, 100, and 200 μg/L) and a solvent control (treated with 0.01% DMSO) of the experimental solution (100 mL) were prepared in 125-mL glass beakers. Ten experimental animals were placed randomly in each of the beakers. Each treatment was made up of three replicates. The experimental conditions were identical to those used during culturing. To keep the CBZ concentrations constant, the test medium in each beaker was renewed daily. The test animals were fed daily with *S. obliquus* to avoid potential stress from starvation. Molting was examined by counting shed carapaces. Survival and molting frequency (number of molts) were recorded daily. The shed carapaces and immobile animals were removed after observation to avoid the potential aggregation of microorganisms in the exposure medium. The exposure medium (50 mL) was sampled before renewing the exposure medium every day and stored at 4 °C prior to chitobiase analysis. 

#### 2.3.2 Chronic Exposure

Chronic toxicity tests (21 days) were performed following the OECD test guideline 211 [28]. Test concentrations for chronic exposure were selected based on the 96 h EC_50_ (median effective concentration) obtained from the acute toxicity tests. The test animals were exposed to four nominal CBZ concentrations (0.03, 0.3, 3, and 30 μg/L (10% of the 96 h EC_50_ of chitobiase)), and treatment with 0.01% DMSO was used as a solvent control. One experimental animal (<24-h-old newborn) was placed randomly in each of the 125-mL glass beakers (contained 100 mL experimental solution). Each treatment was made up of six replicates. The water and CBZ or DMSO was refreshed daily. The experimental conditions were identical to those used during culturing. The duration of the experiment was 21 days, during which survival and molting were monitored daily. Dead individuals were confirmed under a microscope and then removed. Molting was examined by counting shed carapaces [23]. Offspring production was measured daily; once counted, the offspring were removed. The physiological parameters, including the number of molts, the time to first brood of the females, the size of first brood of females, the mean number of broods per female, the number of offspring in each brood, and the total number of offspring per female were recorded to evaluate the toxicity of CBZ on the reproduction in *D. similis*.

During the 21 days of chronic exposure, the number of immobile adults, newborns, and shed carapaces were counted and removed daily. For body length measurements, after the first brood of the females, the surviving individuals were collected separately with a pipette and transferred into a polystyrene cup. Here, they were briefly deposited into a drop of exposure medium and photographed using a CCD (Charge-Coupled Device) (DS-Fi2, Nikon, Tokyo, Japan) fitted to a binocular dissecting microscope (Eclipse Ni, Nikon, Japan) [24]. Surviving adults were then reintroduced into the beakers and fed with green microalgae. Body length was measured on the pictures from the eye to the base of the tail spin using an image analysis software (NIS-elements, Nikon, Tokyo, Japan) [29].

### 2.4. CBZ Quantification

CBZ concentrations were quantified using ultra-performance liquid chromatography tandem mass spectrometry (UPLC–MS/MS; Waters Corporation, Milford, MA, USA) according to a previously described method [6,30] in the analysis and testing center of Nanjing Institute of Geography and Limnology, Chinese Academy of Sciences. Since the test medium in each beaker was renewed daily, CBZ concentrations were quantified immediately after the beakers were dosed only at the first day of the exposure. Briefly, water samples were filtered through a 0.22-μm GH Polypro membrane filter (PALL, New York, NY USA) to remove any suspended matter and injected into a Waters Acquity UPLC fitted with a BEH C18 column (50 × 2.1 mm, 1.7 μm; Waters, Milford, MA, USA) maintained at 30 °C. A tandem Quattro Premier XEV TQD mass spectrometer (Waters, Milford, MA, USA) equipped with an electrospray ionization (ESI) ion source was used for CBZ detection, with all measurements taken in positive ESI mode. The multiple reaction monitoring (MRM) transitions were 237.1 > 194.2 and 237.1 > 179.2 for quantification and confirmation, respectively. The limit of quantification (LOQ) of the method was 0.02 ng/L. The CBZ concentrations determined in all treatments are shown in Table 1. The nominal CBZ values are used throughout the remaining text.

### 2.5. Measurement of Chitobiase Activity 

During the acute exposure, the activities of the release of chitobiase into the water were measured using a commercial kit (Jiancheng, Nanjing, China) according to the manufacturer’s protocol. The chitobiase kit used *p*-nitrophenyl-glucoside as the reaction substrate. When hydrolyzed by N-acetyl-beta-glucosaminidase (NAG), p-nitrophenol was generated from the substrate. Reactions were conducted at 37 °C for 15 min and stopped by the addition of sodium carbonate. Absorbances of the reaction mixtures were read at 400 nm using a spectrophotometer. One unit of chitobiase activity was defined as 1 µmol of *p*-nitrophenol generated per min at 37 °C.

### 2.6. Statistical Analysis

All statistical analyses were performed using SPSS software (version 16.0; SPSS, Chicago, IL, USA) and OriginPro (version 16.0; OriginLab, Northampton, MA, USA) software. The EC_50_ values were calculated by SPSS software based on the probit method. Quantitative data were expressed as mean ± standard error of the mean (*SEM*) in the acute exposure, and the mean ± standard deviation (*SD*) in the chronic exposure. The significance of the differences between the exposed and control samples was determined using a one-way ANOVA (*p* < 0.05) followed by Dunnett’s test for multiple comparisons.

## 3. Results

### 3.1. Acute Toxicity

#### 3.1.1 Acute Toxicity of CBZ on the Molting in *D. similis*

**Figure 2:**
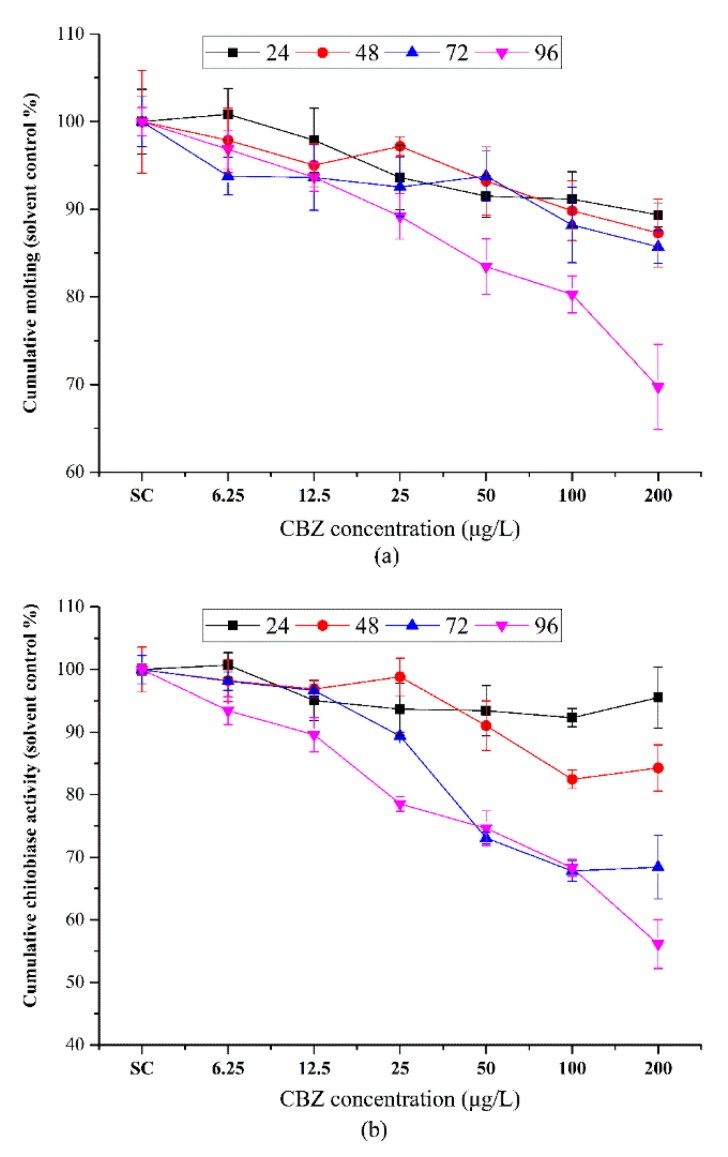
Effects of carbamazepine on cumulative molting (**a**) and chitobiase release (**b**) in juvenile *Daphnia similis* during acute exposure. Results are presented as mean ± *SEM*, *n* = 3 (10 individuals in each replicate).

Over the course of the 4 days acute exposure to various CBZ concentrations, less than 3% mortality was observed at all CBZ concentrations (data not shown) and exposure to lower concentrations of CBZ did not result in any lethality. The results showed that the acute effect of CBZ on *D. similis* was not detected in the current exposure concentration levels. The effect of CBZ on molting frequency of *D. similis* was not concentration-dependent for all the concentrations tested (Figure 2a), with 200 μg/L significantly reducing molting after 48, 72, and 96 h. No marked change in molting frequency was detected after 24 h exposure or at concentrations lower than 6.25 μg/L. Based upon the concentration-response curves (Figure 2) and calculated using the SPSS software based on the probit method, the estimated EC_50_ of CBZ for survival were 864.38 μg/L (96 h) (Table 2).

#### 3.1.2. Acute Toxicity of CBZ on the Chitobiase in *D. similis*

Results from the acute toxicity test showed that the effect of CBZ on the release of chitobiase in *D. similis* was also concentration-dependent, with exposure to 100 μg/L CBZ significantly reducing the release of chitobiase after 48, 72, and 96 h of exposure (Figure 2b). The estimated EC_50_ values of CBZ for the release of chitobiase were 3985.24 μg/L (48 h), 345.58 (72 h), and 306.17 μg/L (96 h). A summary of the estimated effect concentrations for different endpoints is presented in Table 2. 

### 3.2. Chronic Toxicity

#### 3.2.1. Survival and Molting 

**Figure 3:**
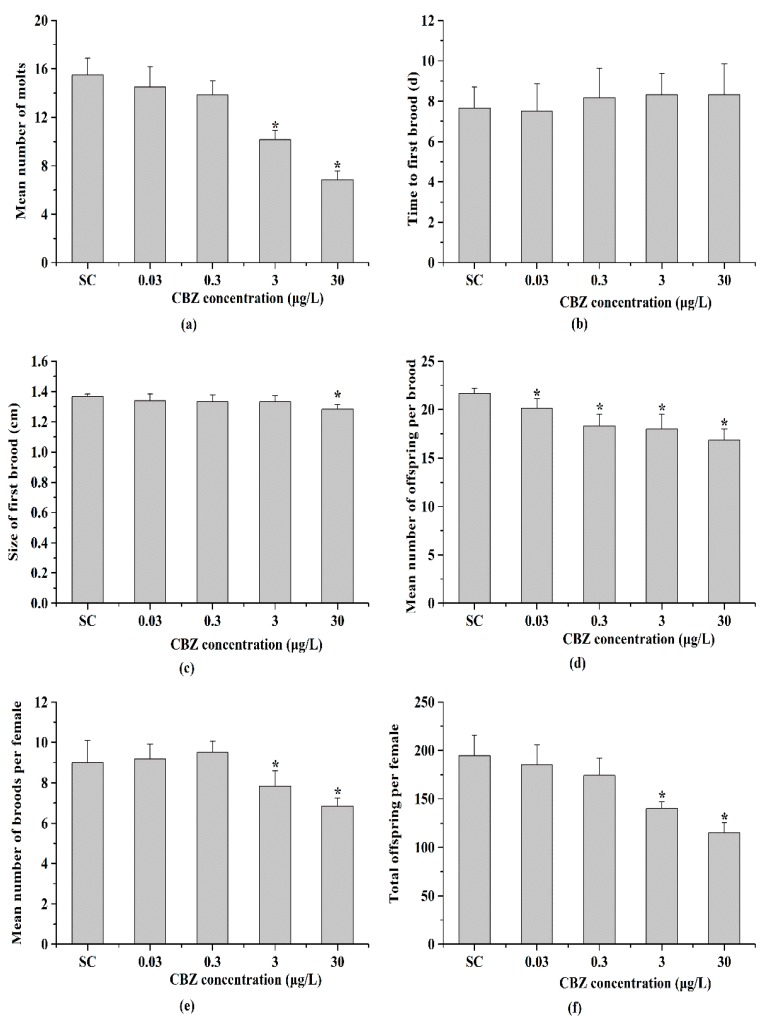
The individual endpoint measurements after the chronic exposure (21 d). (**a**) Mean number of molts; (**b**) Time to first brood (days); (**c**) Size of the first brood (cm); (**d**) Mean number of offspring per brood; (**e**) Mean number of broods per female; (**f**) Total offspring per female. Results are presented as mean ± *SD*, *N* = 6. * *p* < 0.05.

Over the course of the 21 days of exposure to various CBZ concentrations, less than 2% mortality was observed at all CBZ concentrations (data not shown). However, CBZ significantly (*p* < 0.05) affected the molting of *D. similis* at CBZ concentrations higher than 3 μg/L (Figure 3a). The mean number of molts of individual daphnids in the solvent control group was 15.50 ± 1.38. Whereas, the mean number of molts for individual daphnids at 3 and 30 μg/L CBZ was 10.17 ± 0.75 and 6.83 ± 0.75, respectively, which were both significantly decreased compared to the control group (*p* < 0.05; Figure 3a).

#### 3.2.2. Age and Body Size of First Brood

The time to first brood of the females and the size of first brood of females were also measured during the chronic exposure as the two variables of development used to evaluate the effects of CBZ on development in *D. similis*. In the solvent control group, the mean time to first brood of the females was 7.67 ± 1.03 days; no significantly inhibition was observed at the CBZ treated groups ((*p* > 0.05; Figure 3b). On the other hand, another variable of development, the size of first brood of females, was significantly reduced by CBZ only at the highest concentration (30 μg/L) (*p* < 0.05; Figure 3c).

#### 3.2.3. Fecundity

The toxicity of CBZ on the fecundity was evaluated by measuring the mean number of broods per female, the number of offspring in each brood, and the total number of offspring per female throughout the chronic period. These three physiological parameters all decreased with increased CBZ exposure. The mean number of offspring per brood was more sensitive than the other two, with significant inhibition detected at all four of the CBZ exposure groups (*p* < 0.05; Figure 3d). The number of offspring in each brood and the total number of offspring per female were similar, both significantly inhibited only at the CBZ concentrations of 3 and 30 μg/L groups (*p* < 0.05; Figure 3c,d).

## 4. Discussion

*Daphnia* spp., an ecologically important group of model species with short life cycles, strong reproductive abilities, and sensitivity to their chemical environment, has been widely used in aquatic toxicology bioassays for many years [23,31]. Moreover, *Daphnia* play a critical role in aquatic food webs by serving as an intermediate between primary producers and fish; as such, life history changes in *Daphnia* could trigger community- or ecosystem-level responses [31]. In the present study, the small crustacean *D. similis* was used as a model organism to evaluate the acute and chronic toxicity of the pharmaceutical CBZ on the molting and reproduction in crustaceans. *D. similis* was selected because it has a critical role in the freshwater ecosystems of Lake Taihu, China, and has a similar body size with the common model organism *D. magna*. *D. similis* have also been used in field and laboratory studies to measure environmental perturbations or contaminations [24]. Many of the EC_50_ values shown in Table 2 are predicted values, because effects of less than 50% were observed during the acute exposure. Since the standard exposure method and statistical approaches of OECD test guidelines were used, the results are reliable.

### 4.1. Effect of CBZ on the Survival and Molting in D. Similis

After acute exposure to CBZ, no effect was observed on the survival of *D. similis*. It seems that CBZ did not cause acute lethal effects in *D. similis*. Similar to our study, the conclusions of various studies suggested that environmental pharmaceuticals do not cause acute toxic effects [32,33,34]. For example, the 48 h EC_50_ value for *D. magna* was > 13,800 μg/L [33], however, Kim et al. [34] reported that the acute EC_50_ values of CBZ for the same species was >100 mg/L for 48 h and 76.3 mg/L for 96 h [34], whereas over the course of the 21 days exposure to various CBZ concentrations, less than 2% mortality was observed at all CBZ concentrations. The results showed that even the highest concentration of 30 μg/L (10% of the 96 h EC_50_ of chitobiase) was also too low to be lethal. Thus, chronic exposure studies with more specific endpoints other than survival should be used in CBZ risk assessment in daphnids [6,33].

Molting in crustaceans is an important biological process for growth, development, and reproduction [35]. However, to our knowledge, studies involving the toxicity of CBZ on molting in crustaceans is limited. The molting of *D. similis* can be inhibited by many environmental contaminants, such as ammonia and nitrite, as well as hypoxia [23,24]. These subtle, sublethal effects on the normal life processes of *Daphnia* often occur at concentrations that are several orders of magnitude lower than lethal concentrations. It seems that molting was more sensitive than immobility, thus it would be a good endpoint to detect the acute toxicity of CBZ in crustaceans. Moreover, the chronic exposure also proved the sensitivity of molting. CBZ significantly affected the molting of *D. similis* at CBZ concentrations higher than 3 μg/L after the 21 days of chronic exposure. Although 3 μg/L is just a little higher than the concentration at which CBZ is found in the environment, more attention should be paid to molting rather than survival when CBZ toxicity is considered.

### 4.2. Toxicity of CBZ on the Release of Chitobiase in D. Similis

In the process of molting, arthropods living in water will release chitobiase-rich peeling liquid into water. The larger the number and size of the aquatic arthropods, the higher the release of chitobiase in vitro [36]. Therefore, theoretically, chitobiase can be a prerequisite for biomass indicators of aquatic arthropods. The relationship between chitobiase release in vitro and the biomass of aquatic arthropods has been confirmed in some laboratory experiments [21]; the results of field investigations also confirm the existence of this relationship to some extent [37]. In the current study, after acute exposure to CBZ, the release of chitobiase in *D. similis* was concentration-dependent during the 4-day exposure period. Compared with the control, CBZ significantly reduced the release of chitobiase. Moreover, with the decrease in chitobiase, molting of *D. similis* was also inhibited. This shows that chitobiase activity should be an indicator of altered growth and reproduction in *D. similis* exposed to CBZ. Similar results were also reported by Duchet et al.; after exposed to spinosad and diflubenzuron, the chitobiase activity acted as an indicator of altered survival, growth, and reproduction in *Daphnia pulex* and *D. magna* [38]. 

### 4.3. Chronic Toxicity of CBZ on the Reproduction in D. similis

Previous studies have suggested that CBZ could act as an endocrine disruptor in *Daphnia*, as it decreases their reproductive output and causes developmental abnormalities in their offspring [19]. Therefore, the chronic toxicity of CBZ on the reproduction in *D. similis* was tested in the current study. As described above, the chitobiase activity is an indicator of altered reproduction in *Daphnia* [38], so 10% of the 96 h EC_50_ of chitobiase was referred to for selecting the concentrations of the chronic exposure. After chronic exposure, the endpoints showed that the reproduction of *D. similis* was significantly inhibited by CBZ. Chronic exposure to CBZ resulted in smaller size of the first brood of the females, reduced fecundity (the mean number of broods per female, the number of offspring in each brood, and the total number of offspring per female). These subtle, sublethal effects on the normal life processes of *Daphnia* often occur at concentrations that are several orders of magnitude lower than the lethal concentrations [39]. For example, Oropesa et al. showed that chronic exposure to CBZ significantly decreased the reproductive output and number of *D. magna* at 200 μg/L [40], which was similar to our result. A previous study reported that aromatic amino acids, including serine, glycine, and alanine, of *D. magna* were potential bioindicators for sub-lethal CBZ exposure that may have altered their metabolism [20]. Therefore, the alteration of energy metabolism in *D. similis* by CBZ may lead to the changes in the molting and reproduction. On the other hand, opposite results were reported by Rivetti et al., who showed that, as a neuro-active pharmaceutical, CBZ was able to enhance reproduction at 1 μg/L of CBZ [19]. The different results may be due to the higher concentrations used in the current study. To resolve this, further research should be conducted using a wide range of concentrations. 

The results of the chronic exposure together with the acute exposure suggested that CBZ acts as an endocrine disruptor in *D. similis*. Further, when comparing the effect concentrations of CBZ between different endpoints among the acute and chronic exposures, the release of chitobiase was identified to be the most sensitive endpoint in response to CBZ. The LOEC obtained from the current study suggested that chitobiase was the most sensitive indicator of changes to CBZ. The expression of chitobiase is positively regulated by the endogenous molting hormone 20-hydroxyecdysone through transcriptional activation of the ecdysone receptor (EcR) [22,41]. Even it is still not clear how release of chitobiase correlates with the molting [21], the results of the current study suggest that CBZ acts as an EcR agonist in *D. similis*. Future relevant toxicity tests, especially chronic toxicity tests with multiple endpoints, such as chitobiase mRNA, are expected to enrich the toxicity test database of the toxicity of CBZ in *Daphnia*. 

## 5. Conclusions

In the present study, we evaluated the acute and chronic aquatic toxicity of the most frequently detected pharmaceutical compound (CBZ) in aquatic environments, at environmentally relevant concentrations, using the model organism *D. similis*. The acute toxicity results showed that CBZ did not cause lethal toxicity at the tested concentrations. However, significant effects of CBZ on the molting and fecundity in *D. similis* were observed even at concentrations as low as 0.03 μg/L. Our results show that CBZ toxicity to *D. similis* depends on the timing and duration of the exposure. In conclusion, CBZ can cause inhibition of molting, delayed reproduction, and reduced fecundity in *D. similis*. Moreover, since the release of chitobiase is an indicator of potential molting and reproductive disruption, CBZ acts as an endocrine disruptor in *D. similis*, as with vertebrates (e.g., fish). It is expected that CBZ exposure may lead to a broad range of developmental and reproductive endpoints in invertebrate populations, and thus further research on the ecological impacts of CBZ should be conducted. 

## Figures and Tables

**Table 1:** Carbamazepine (CBZ) concentration (mean ± *SD*) measured in the water of the acute and chronic exposure experiment.

Exposure	Treatments	NC ^1^ (μg/L)	MC ^2^ (μg/L)
Acute exposure	Solvent Control	0	<LOQ ^3^
	6.25 μg/L	6.25	6.25 ± 0.05
	12.5 μg/L	12.5	12.62 ± 0.21
	25 μg/L	25	25.68 ± 0.15
	50 μg/L	50	50.26 ± 1.25
	100 μg/L	100	100.89 ± 2.63
	200 μg/L	200	203.40 ± 2.59
Chronic exposure	Solvent Control	0	<LOQ ^3^
	0.03 μg/L	0.03	0.03 ± 0.01
	0.3 μg/L	0.3	0.30 ± 0.06
	3 μg/L	3	3.02 ± 0.53
	30 μg/L	30	31.86 ± 0.88

^1^ Nominal Concentrations; ^2^ Measured Concentrations; ^3^ Limit of Quantification.

**Table 2:** Summary of the effect concentrations (μg/L) of carbamazepine for different endpoints in juvenile *Daphnia similis* during acute exposure.

Time	Endpoint					
	Molting			Chitobiase		
	NOEC ^1^	LOEC ^2^	EC_50_^3^	NOEC	LOEC	EC_50_^3^
24 h	200	>200	/ ^4^	200	>200	/ ^4^
48 h	100	200	/ ^4^	50	100	3985.24^5^
72 h	100	200	/ ^4^	12.5	25	345.58^5^
96 h	12.5	25	864.38 ^5^	<6.25	6.25	306.17^5^

^1^ No Observed Effect Concentration; ^2^ Lowest Observed Effect Concentration; ^3^ Median Effective Concentrations; ^4^ No values were calculated based on the probit method; ^5^ Predicted values, observed effect less than 50%.
